# The Adaptation Time to the Extender as a Crucial Step for an Accurate Evaluation of Ram Sperm Quality during the Liquid Storage

**DOI:** 10.3390/vetsci11030132

**Published:** 2024-03-16

**Authors:** Marta Neila-Montero, Mercedes Alvarez, Marta F. Riesco, Cristina Soriano-Úbeda, Rafael Montes-Garrido, Cristina Palacin-Martinez, Paulino de Paz, Luis Anel, Luis Anel-Lopez

**Affiliations:** 1 Itra-ULE, INDEGSAL, University of León, 24071 León, Spain; mneim@unileon.es (M.N.-M.); mferrs@unileon.es (M.F.R.); msoru@unileon.es (C.S.-Ú.); rmong@unileon.es (R.M.-G.); cpalm@unileon.es (C.P.-M.); ppazc@unileon.es (P.d.P.); laner@unileon.es (L.A.); lanel@unileon.es (L.A.-L.); 2 Animal Reproduction and Obstetrics, Department of Veterinary Medicine, Surgery and Anatomy, University of León, 24071 León, Spain; 3 Cellular Biology, Department of Molecular Biology, University of León, 24071 León, Spain; 4 Anatomy, Department of Veterinary Medicine, Surgery and Anatomy, University of León, 24071 León, Spain

**Keywords:** cooled sperm, membrane stability, ovine, semen preservation, stabilization

## Abstract

**Simple Summary:**

This study focused on the importance of accurately assessing ram sperm quality for optimizing assisted reproductive technologies in sheep. Semen preservation can lead to sperm damage due to various stressors, including osmotic, biochemical, and thermal factors. To address this issue, the research aimed to determine the best time to evaluate ram sperm quality and identify the factor causing changes in sperm quality during liquid storage. In *Experiment 1*, ejaculated sperm were assessed for motility and functionality at different preservation times: 0, 3, 6, and 24 h. Both motility and functionality improved after 6 h. *Experiment 2* delved into the responsible factor by using epididymal sperm. Results revealed that extender addition to the sperm caused altered motility at 0 and 24 h, and reduced functionality at 0 h. This suggests that the extender initially alters ram sperm, leading to sublethal damage that becomes reversible after 3 to 6 h of semen preservation. Thus, ram sperm require an adaptation period of 3 to 6 h to the extender before a precise quality assessment. This finding has practical implications for reproduction centers, allowing better workflow organization and optimal expression of ram sperm attributes at the time of cervical artificial insemination.

**Abstract:**

Accurate assessment of ram sperm quality is crucial to optimizing assisted reproductive technologies in sheep. However, semen preservation can induce sperm due to osmotic, biochemical, and thermal stress. Stabilizing sperm with a suitable cooling rate and adaptation period to the extender could mitigate these effects for a more reliable evaluation. This study aimed to determine: (1) the best time to assess ram sperm quality, and (2) the factor responsible for the altered state of ram sperm during the first hours of liquid storage. In *Experiment 1*, ejaculated sperm were diluted and assessed for sperm motility and functionality at four preservation times: 0, 3, 6, and 24 h as sperm damage control. Both sperm motility and functionality improved after 6 h. *Experiment 2* investigated the factor responsible for sperm quality change by testing the interactions of seminal plasma and extender with sperm from epididymides independently and in combination. The evaluation of sperm was performed as in *Experiment 1*. Sperm in groups containing the extender showed altered motility at 0 and 24 h, and lower functionality at 0 h. Thus, we could assume that extender addition initially alters ram sperm, causing sublethal damage that is reversible after 3 to 6 h of semen preservation. In conclusion, ram sperm require an adaptation time of 3 to 6 h to the extender before an accurate quality assessment can be conducted. This has practical implications for reproduction centers, enabling better workflow organization and optimal expression of ram sperm attributes when cervical artificial insemination is routinely performed.

## 1. Introduction

Ovine livestock has notably increased benefits in recent decades due to the improvements in reproductive performance and genetic gain achieved through the implementation of assisted reproductive technologies (ART) such as semen preservation, estrus synchronization, artificial insemination (AI), and in vitro embryo production, development, and transfer [1,2]. The high efficiency of these techniques mainly depends on the quality of the semen used [3], so it is important to perform a precise evaluation of sperm quality in order to select males with good fertilizing potential, discarding those cases of infertility or subfertility [4]. Unfortunately, predicting the potential fertility of a sperm sample is still considered a utopian endeavor due to the inaccurate information provided by the sperm laboratory assays and the unreliability of the fertility data used for establishing correlations with the outcomes of in vitro tests [5]. Semen samples are generally processed after collection to prolong the fertile lifespan of sperm by depressing or completely arresting their metabolism using reduced storage temperatures in either a liquid or cryopreserved state [6]. However, despite the large number of studies on the diluents used [7,8,9], cooling and freezing protocols [10,11,12], and sperm storage concentration [13,14,15], the processing and storage of ram semen can still induce sublethal damage to sperm that make them behave very differently from fresh sperm [6]. During semen preservation, sperm are exposed to artificial conditions leading to a certain degree of osmotic, biochemical, and thermal stress [16,17]. These stressors compel sperm to adapt to a hostile environment by means of conformational and metabolic changes, resulting in membrane redistribution, lipid peroxidation, and altered ATP generation and motility [18]. Thus, the stabilization of sperm with an adequate cooling rate coupled with a suitable adaptation period to the extender would enable them to overcome the damaging effects of the different stress factors to which they are subjected in semen preservation [19]. There are many studies on the impact of this adaptation time on the cryoresistance of sperm from several species, such as dogs [20,21], boars [22,23,24], bulls [25,26,27,28], bucks [29], and rams [30,31,32,33,34], with different outcomes. For ram sperm, in general, an adaptation period of 2 to 8 h seems to be sufficient to ensure greater post-thawing sperm quality [35,36], but we have also noticed that the adaptation time to the extender during liquid storage influences sample quality. Our research group has long observed that conducting sperm quality evaluations close to the collection time gives inaccurate results, as quality improves over time. Although the composition of the extender impacts sperm survival during cooled storage, INRA 96^®^ contains a highly protective fraction: native phosphocainate (NPPC) [37]. Nevertheless, there is no consensus on the effect of seminal plasma on sperm function. On the one hand, some studies have demonstrated the benefits of the removal of seminal plasma, deemed a sperm membrane destabilizing factor by Barrier-Battut and colleagues [38], before liquid storage in several species [39,40,41,42]. On the other hand, other authors have not seen any advantage of seminal plasma removal during cooled preservation [43,44].

Thus, this study aimed to establish the best time to perform an optimal assessment of the sperm quality in ram semen samples preserved at 15 °C for up to 6 h, as well as to determine the factor responsible for the altered state of sperm during the first hours of liquid storage using epididymal sperm to test interactions with the two possible factors in an independent way: seminal plasma and extender.

## 2. Materials and Methods

### 2.1. Animals

A total of 16 healthy, mature, and proven fertile Assaf rams trained for semen collection by artificial vagina (weekly semen collections, twice per week) participated in the experiments. Rams were housed at the Animal Selection and Reproduction Center of Junta de Castilla y León (CENSYRA, Villaquilambre, León, Spain), and fed a balanced diet standardized for the species and age.

### 2.2. Study Design

#### 2.2.1. Experiment 1: Establishment of a Stabilization Period for Ram Sperm

**Figure 1:**
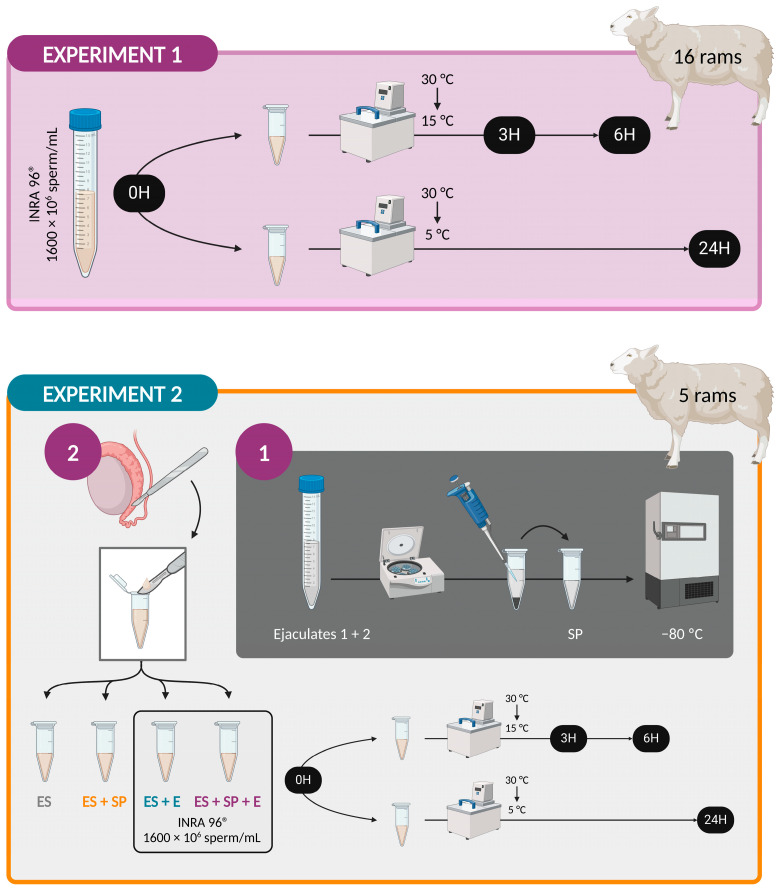
Study design. *Experiment 1:* Establishment of a stabilization period for ram sperm through sperm quality evaluation at four preservation times: 0 h at 30 °C, 3 and 6 h at 15 °C, and 24 h at 5 °C. *Experiment 2:* Identification of the factor altering ram sperm quality through sperm quality evaluation in four experimental groups: epididymal sperm (ES), epididymal sperm with seminal plasma (ES + SP), epididymal sperm with INRA 96^®^ (ES + E), and epididymal sperm with seminal plasma and INRA 96^®^ (ES + SP + E). Created with BioRender.com.

One ejaculate from 16 males was collected using an artificial vagina at 40 °C (IMV Technologies, L’Aigle, France) with the aid of a female decoy during the reproductive season. Semen tubes were initially placed in a tempered bath (30 °C) for quality evaluation, which included: (i) ejaculate volume determination by using the graduation marks on the collection tube, (ii) mass motility analysis on a subjective score from 0 to 5 under a microscope (Leica DM LB, Meyer Instruments, Houston, TX, USA) at 40× magnification and on a stage warmed at 37 °C, and (iii) sperm concentration assessment with a cell-counting device (NucleoCounter SP-100, ChemoMetec, Allerod, Denmark). Only ejaculates that met the minimum requirements of a volume of 0.5 mL, mass motility of 4, and sperm concentration of 3 × 10^9^ sperm/mL were used in this study and diluted in INRA 96^®^ medium (NPPC-based extender containing potassium penicillin G, gentamicin, and amphotericin B; IMV Technologies, L’Aigle, France) up to 1600 × 10^6^ sperm/mL. High-quality ejaculates were then transported to our laboratory at the University of León (about a 10 min trip), with the arrival time representing the time of 0 h at which sperm quality is classically assessed. At this point, samples were split into two equal aliquots and refrigerated in a programmable bath (CC-K8, Huber, Germany). One aliquot of each sample was cooled at −0.5 °C/min from 30 °C to 15 °C and preserved at 15 °C for 3 and 6 h. The other aliquot was subjected to a cooling rate of −0.25 °C/min from 30 °C to 5 °C and stored at 5 °C for 24 h, serving as positive damage control in our sperm preservation protocol (Figure 1).

#### 2.2.2. Experiment 2: Identification of the Factor Altering Ram Sperm Quality

During the breeding season, two ejaculates were collected from 5 males by using an artificial vagina. Both ejaculates from each male were mixed and centrifuged at 4 °C for 15 min at 10,000× *g*. Seminal plasma was collected, checked for purity using phase-contrast microscopy, and kept at −80 °C until use. Rams were slaughtered at the slaughterhouse the week following the collection of seminal plasma. Their testicles were transported to our laboratory in a portable refrigerator at 22 °C (CoolFreeze CF-25, Dometic Group, Stockholm, Sweden). On arrival, about 30 min post-mortem, epididymides were dissected and cleared of connective tissue and surface blood vessels to prevent blood contamination of the samples. Sperm was then collected by making several incisions in the cauda epididymis and removing the emerging fluid with a surgical blade. Epididymal sperm were split at this moment into four aliquots to form four different experimental groups in order to test interactions with seminal plasma and extender in an independent way, doubling each of the tubes. The first experimental group contained only epididymal sperm (ES). The second simulated a physiological ejaculate with the addition of 30% autologous seminal plasma (*v*/*v*) to the epididymal sperm (ES + SP). The third one was generated with the dilution of the epididymal sperm to obtain a concentration of 1600 × 10^6^ sperm/mL in INRA 96^®^ (ES + E). The last experimental group was obtained with the addition of 30% autologous seminal plasma and INRA 96^®^ to the epididymal sperm until a final concentration of 1600 × 10^6^ sperm/mL was achieved in an attempt to simulate an ejaculate processed in the usual form (*Experiment 1*) (ES + SP + E). This represented the time of 0 h. Afterward, one replicate of the samples was cooled in a programmable bath at a rate of −0.5 °C/min from 30 °C to 15 °C and stored at 15 °C for 3 and 6 h. The other set of samples was refrigerated at −0.25 °C/min from 30 °C to 5 °C and stored at 5 °C for 24 h (Figure 1). Autologous seminal plasma (from the same ram that provided the sperm) was used to minimize interference with the results, as several authors have reported variations in seminal plasma composition among species, among males of the same species, and even among ejaculates from the same male [45,46,47,48,49].

### 2.3. Sperm Quality Evaluation

#### 2.3.1. Sperm Motility and Kinetic Parameter Analysis with the CASA System

Sperm motility and kinetic parameters were assessed with Computer-Assisted Sperm Analysis (CASA) using Sperm Class Analyzer^®^ (SCA) software version 6.3.0.59 (Microptic S.L., Barcelona, Spain). The system settings comprised recording 50 frames at 100 frames/s and following the trajectory of particles in an area ranging from 20 to 70 µm^2^. Samples were diluted up to 25 × 10^6^ sperm/mL in a TES-Tris-Fructose medium with 1% clarified egg yolk (320 mOsm/kg, pH 7.2) and pre-warmed for 5 min at 37 °C. A 5 µL drop from each sample was then placed in a Makler chamber (depth of 10 µm; Sefi Medical Instruments, Haifa, Israel) and analyzed on a warmed stage (37 °C) at 100× magnification with a phase-contrast microscope (Eclipse E400, Nikon, Tokyo, Japan) and a BASLER acA1300-200uc digital camera (Basler Vision Technologies, Ahrensburg, Germany). Four random fields were captured and subsequently analyzed by removing non-sperm events. The kinetic parameters obtained were curvilinear velocity (VCL, μm/s), linearity (LIN, %), and amplitude of lateral sperm head displacement (ALH, μm). Total motility (TM), progressive motility (PM), and fast progressive motility (FPM) were referred to as the proportion of sperm with VCL > 15 μm/s, 45 μm/s, and 75 μm/s, respectively.

#### 2.3.2. Sperm Functionality Analysis with Flow Cytometry

Samples in the different experimental groups were first washed with a brief centrifugation spin for 15 s (MiniSpin plus, Eppendorf, Hamburg, Germany) in phosphate-buffered saline (PBS) (300 mOsm/kg, pH 7.2) at a concentration of 2 × 10^6^ sperm/mL. After the supernatant was discarded, the sperm pellet was stained for 30 min in the dark, as described by Riesco et al. [50]. Plasma membrane integrity was evaluated using the Zombie Violet™ Fixable Viability Kit (BioLegend, San Diego, CA, USA) at 1:1000 in PBS. Sperm apoptosis was determined with CellEvent™ Caspase-3/7 Green Detection Reagent (ThermoFisher, Waltham, MA, USA) at 4 µM in PBS. The content of reactive oxygen species was evidenced with CellROX™ Deep Red Reagent (Invitrogen, Eugene, OR, USA) at 5 µM in PBS. Afterward, a new wash was performed as described above to prevent overstaining, and the cells were resuspended in 1 mL of PBS for analysis in a MACSQuant Analyzer 10 (Miltenyi Biotech, Bergisch Gladbach, Germany) equipped with violet, blue, and red lasers operating at 405, 488, and 635 nm, respectively, and ten photomultiplier tubes. Detection of violet, green, and red fluorescence was conducted on the V1 (excitation 405 nm, emission 450/50 nm), B1 (excitation 488 nm, emission 525/50 nm), and R1 channels (excitation 635 nm, emission 655–730 nm (655LP + split 730)). A total of 40,000 events were recorded per sample at a flow rate of 200 to 300 cells/s. The analysis of the data was performed with FlowJo™ version 10.8.1 (Ashland, Wilmington, DE, USA). The signal of the events was plotted as the percentage of viable sperm (indicated by low-intensity Zombie Violet™ staining), apoptotic sperm (marked by CellEvent™ Caspase-3/7 positivity), and sperm with high mitochondrial activity (identified through positive CellROX™).

### 2.4. Statistical Analysis

Statistical analyses of the data were conducted with the SAS/STAT^®^ statistical package version 9.1 (SAS Institute, Cary, NC, USA), while Prism 9 (GraphPad Software, San Diego, CA, USA) was utilized for generating graphs. Variables were examined for normality, and data with a normal distribution were analyzed with a mixed linear model (MIXED procedure). Each experimental group included the same males. Results are reported as the mean ± SEM (Standard Error of the Mean). Statistical significance was considered when *p* < 0.05.

## 3. Results

### 3.1. Experiment 1: Establishment of a Stabilization Period for Ram Sperm

#### 3.1.1. Sperm Motility and Kinetic Parameters

**Figure 2:**
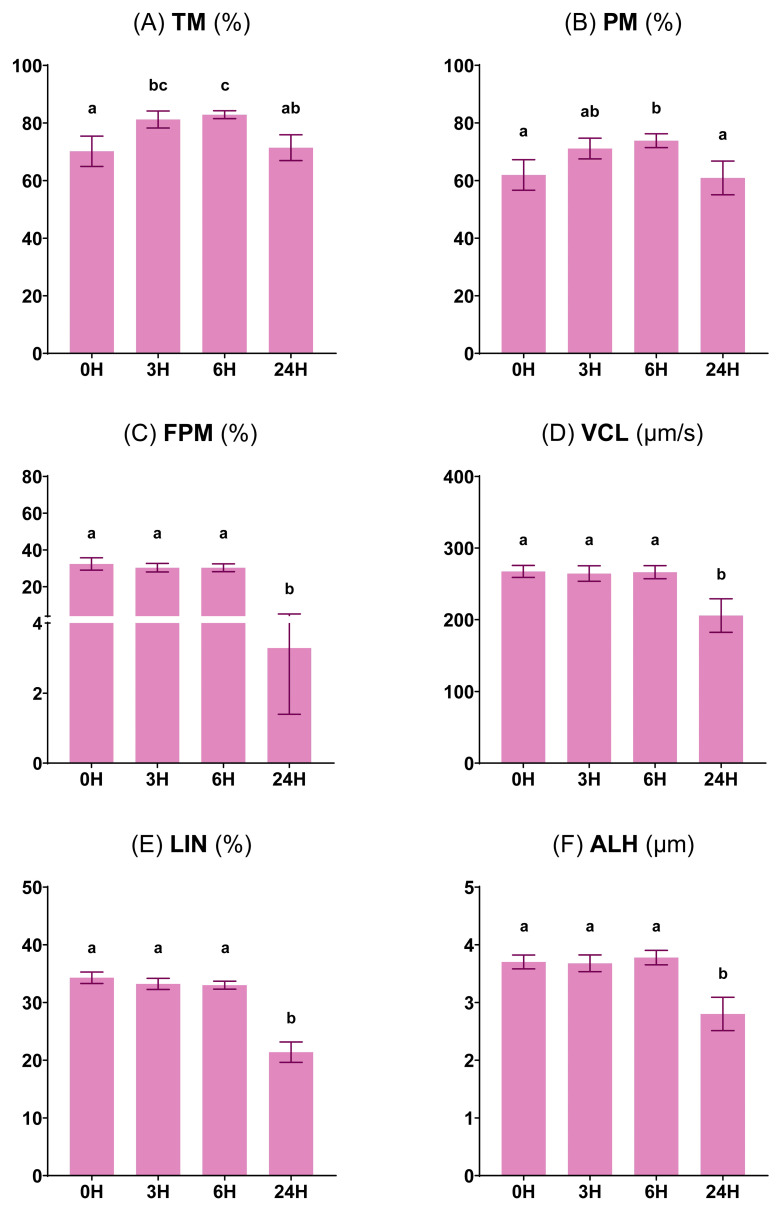
Characteristics of motility and kinetics of ram sperm at four preservation times: 0 h at 30 °C, 3 and 6 h at 15 °C, and 24 h at 5 °C. (**A**) Total motility (TM, %). (**B**) Progressive motility (PM, %). (**C**) Fast progressive motility (FPM, %). (**D**) Curvilinear velocity (VCL, µm/s). (**E**) Linearity (LIN, %). (**F**) Amplitude of lateral head displacement (ALH, µm). Parameters were determined in the same 16 males at each preservation time. Distinct lowercase letters (a–c) denote significant differences (*p* < 0.05) among the values recorded at different preservation times.

Regarding sperm motility measurements, both TM and PM were significantly increased (*p* < 0.05) at 6 h in comparison to the 0 h, with no changes observed (*p* ≥ 0.05) between the 0 and 24 h values (Figure 2A,B). On the other hand, FPM, VCL, LIN, and ALH followed a similar distribution during the whole preservation protocol. All of them were unchanged (*p* ≥ 0.05) after 6 h of storage at 15 °C, but they decreased (*p* < 0.05) after 24 h of refrigeration at 5 °C (Figure 2C–F).

#### 3.1.2. Sperm Functionality

**Figure 3:**
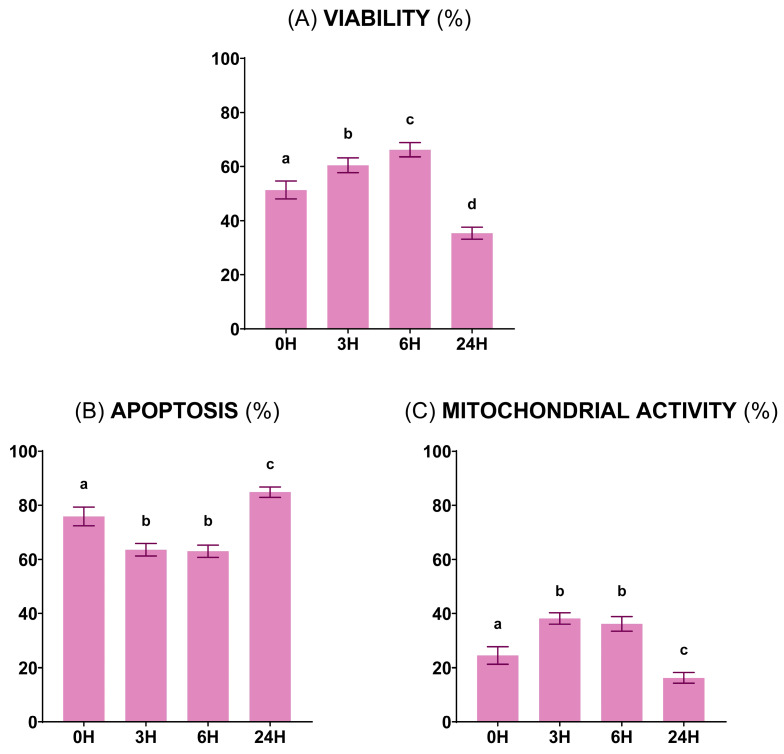
Ram sperm functionality at the four preservation times: 0 h at 30 °C, 3 and 6 h at 15 °C, and 24 h at 5 °C. (**A**) Viable sperm (%) (Zombie Violet™). (**B**) Apoptotic sperm (%) (CellEvent™ Caspase-3/7 Green). (**C**) Sperm with high mitochondrial activity (%) (CellROX™ Deep Red). Parameters were determined in the same 16 males at each preservation time. Distinct lowercase letters (a–d) denote significant differences (*p* < 0.05) among the values recorded at different preservation times.

The results of the sperm functionality analysis revealed that sperm viability increased (*p* < 0.05) from 0 to 3 h of preservation and again after 6 h and decreased significantly (*p* < 0.05) at 5 °C for 24 h, reaching minimum values (Figure 3A). Meanwhile, apoptosis showed the lowest level (*p* < 0.05) at 3 h of storage at 15 °C, which was maintained for up to 6 h. Contrarily, 24 h samples displayed the highest (*p* < 0.05) apoptosis (Figure 3B). Mitochondrial activity demonstrated an opposite pattern to apoptosis, being highest (*p* < 0.05) at 3 and 6 h and lowest (*p* < 0.05) at 24 h (Figure 3C).

### 3.2. Experiment 2: Identification of the Factor Altering Ram Sperm Quality

#### 3.2.1. Sperm Motility and Kinetic Parameters

**Figure 4:**
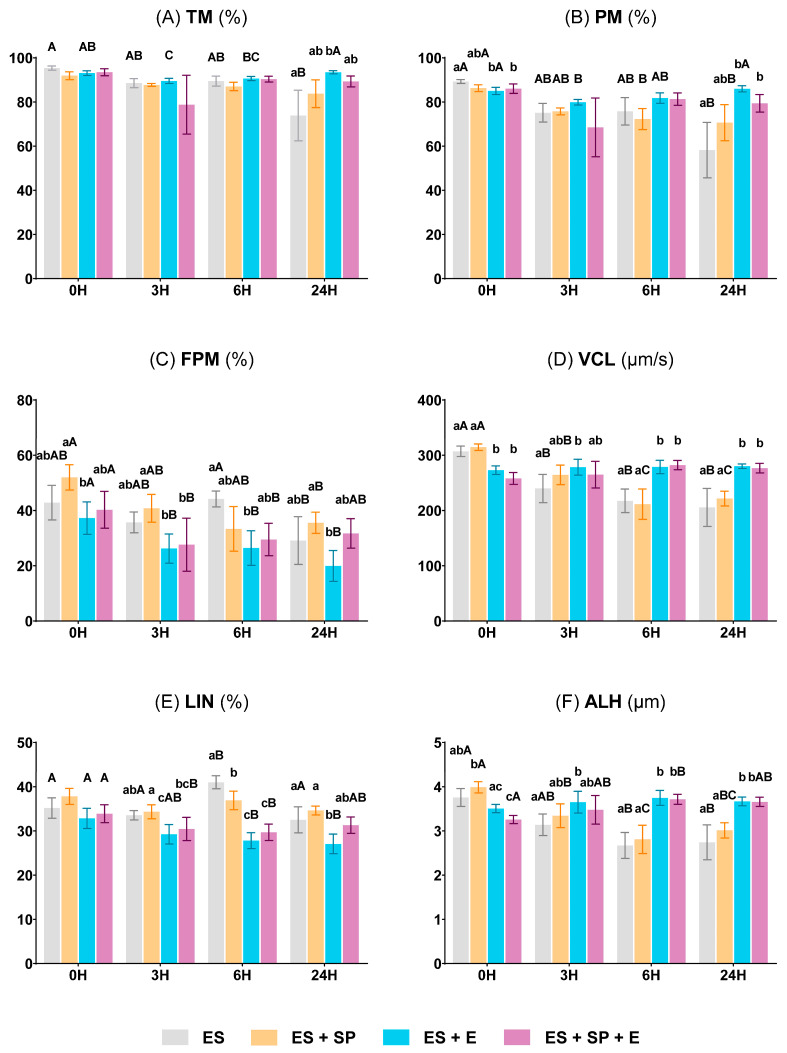
Ram sperm motility and kinetic characteristics in the four experimental groups: epididymal sperm alone (ES), epididymal sperm mixed with seminal plasma (ES + SP), epididymal sperm combined with INRA 96^®^ (ES + E), and epididymal sperm with both seminal plasma and INRA 96^®^ (ES + SP + E). (**A**) Total motility (TM, %). (**B**) Progressive motility (PM, %). (**C**) Fast progressive motility (FPM, %). (**D**) Curvilinear velocity (VCL, µm/s). (**E**) Linearity (LIN, %). (**F**) Amplitude of lateral head displacement (ALH, µm). The measurements were performed in the same 5 males in each experimental group at different preservation times: 0 h at 30 °C, 3 and 6 h at 15 °C, and 24 h at 5 °C. Distinct lowercase letters (a–c) denote significant differences (*p* < 0.05) among the experimental groups at each preservation time, while uppercase letters (A–C) highlight significant differences (*p* < 0.05) among the values recorded at the preservation times within each experimental group.

Figure 4 shows the motility and kinetic characteristics of ram sperm. TM was similar (*p* ≥ 0.05) in the different experimental groups along the preservation protocol at 15 °C for 6 h. However, this parameter was significantly higher (*p* < 0.05, lowercase letters) in ES + E (blue bars) than in ES (grey bars) in the samples stored at 5 °C for 24 h (Figure 4A). PM, VCL, and ALH exhibited significant differences across the experimental groups at 0 h, with markedly lower values (*p* < 0.05, lowercase letters) in the two experimental groups containing INRA 96^®^ (ES + E and ES + SP + E, blue and purple bars, respectively) than in ES (grey bars) (Figure 4B,D,F). The group that simulated the ejaculate (ES + SP + E, purple bars) showed a significant increase (*p* < 0.05, uppercase letters) in ALH at 6 h (Figure 4F). Nevertheless, PM, VCL, and ALH were significantly higher (*p* < 0.05, lowercase letters) in both experimental groups with the extender (ES + E and ES + SP + E, blue and purple bars, respectively) after 24 h of storage at 5 °C, coinciding with a decrease (*p* < 0.05, uppercase letters) compared to the 0 h in the ES and ES + SP groups (grey and orange bars, respectively) (Figure 4B,D,F). On the other hand, FPM was significantly lower (*p* < 0.05, lowercase letters) in ES + E (blue bars) than in ES + SP (orange bars) at both 0 and 24 h (Figure 4C). Finally, LIN progressively decreased in the ES + E group (blue bars) until 6 h of storage at 15 °C, from there it was significantly lower (*p* < 0.05, uppercase letters) (Figure 4E).

#### 3.2.2. Sperm Functionality

**Figure 5:**
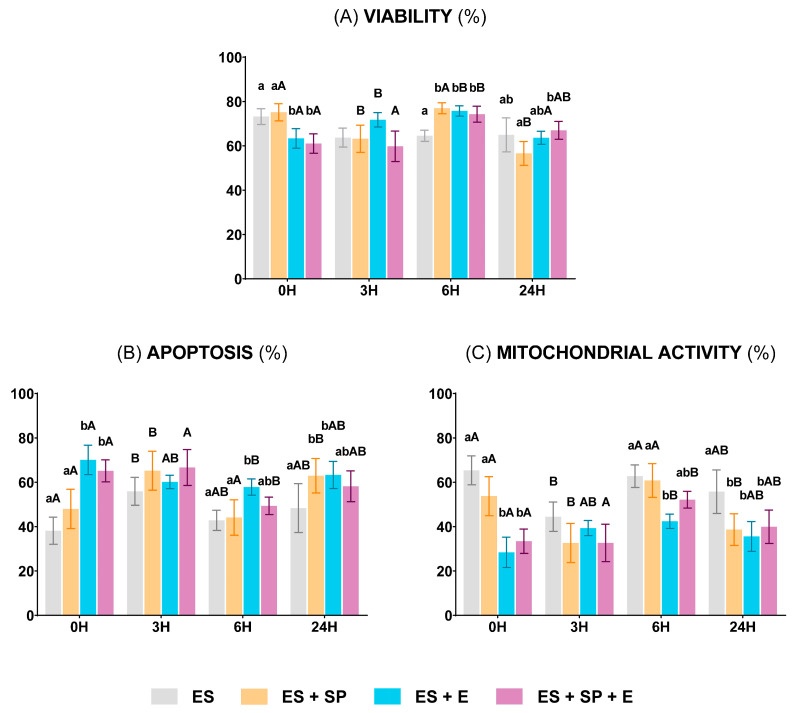
Ram sperm functionality in the four experimental groups: epididymal sperm alone (ES), epididymal sperm mixed with seminal plasma (ES + SP), epididymal sperm combined with INRA 96^®^ (ES + E), and epididymal sperm with both seminal plasma and INRA 96^®^ (ES + SP + E). (**A**) Viable sperm (%) (Zombie Violet™). (**B**) Apoptotic sperm (%) (CellEvent™ Caspase-3/7 Green). (**C**) Sperm with high mitochondrial activity (%) (CellROX™ Deep Red). The measurements were performed in the same 5 males in each experimental group at different preservation times: 0 h at 30 °C, 3 and 6 h at 15 °C, and 24 h at 5 °C. Distinct lowercase letters (a, b) denote significant differences (*p* < 0.05) among the experimental groups at each preservation time, while uppercase letters (A, B) highlight significant differences (*p* < 0.05) among the values recorded at the preservation times within each experimental group.

The most remarkable results of the sperm functionality analysis among the different experimental groups exhibited significantly lower (*p* < 0.05, lowercase letters) sperm viability and mitochondrial activity and significantly higher (*p* < 0.05, lowercase letters) apoptosis in both groups containing the extender (ES + E and ES + SP + E, blue and purple bars, respectively) at 0 h. The evolution during the preservation of these two experimental groups showed that sperm viability and mitochondrial activity increased (*p* < 0.05, uppercase letters) and apoptosis decreased (*p* < 0.05, uppercase letters) after 6 h at 15 °C (Figure 5A–C). Despite this, the ES + E group showed higher (*p* < 0.05, lowercase letters) apoptosis and lower (*p* < 0.05, lowercase letters) mitochondrial activity than the experimental group composed of just epididymal sperm (ES, grey bars) both after 6 and 24 h (Figure 5B,C).

## 4. Discussion

Semen preservation is a critical ART that facilitates the transportation of sperm over long distances and increases the number of females that can be inseminated per ejaculate, ultimately accelerating genetic gain [51]. Ideally, semen preservation must preserve the sperm’s ability to fertilize from ejaculation to use, which is usually assessed on the basis of the results of several laboratory tests on sperm quality [52,53]. In the last decade, increasingly sophisticated, highly quantitative, repeatable, and sensitive methods have been developed to achieve more accurate sperm quality assessments in order to eliminate clear cases of male infertility or subfertility and select ejaculates with high fertilizing potential for use [4]. The most advanced laboratory assays include CASA system motility analysis and a variety of fluorescent dyes for fluorescence microscopy and/or flow cytometry techniques [54,55,56]. However, despite the use of these novel evaluation methods, our research group has long observed that sperm quality assessment close to the collection time is inaccurate, as quality improves over time. After collection, semen is diluted in different extenders depending on the species, use, temperature, and storage duration required [53,57]. The choice of an appropriate extender plays an essential role in minimizing the harmful effects associated with semen preservation, as the diluent is responsible for protecting sperm from osmotic and thermal shock by maintaining an adequate pH and providing buffering capacity [18]. Conflicting results have been reported on the suitability of different extenders for ram semen [58], although Tris and skimmed milk-based are the most commonly used [59]. INRA 96^®^ employed in this work is the main diluent for the preservation of cooled ram semen in Mediterranean countries [9], since NPPC (α, β, and κ caseins) was found to be the most effective component for prolonging the fertile lifespan of cold-stored sperm by reducing the lipid loss from the sperm membrane [60,61]. For liquid preservation, ram semen is progressively cooled after dilution from collection to storage temperature (15 °C or 5 °C). The detrimental impact of cooling ungulate semen (particularly through a rapid decrease from 30 °C to 5 °C) on sperm viability has been widely documented, being proportional to the cooling rate and temperature range involved [62]. Although this adverse effect, referred to as cold shock, predominantly occurs right after ejaculation since the cells become less sensitive in the following hours [63], it was discarded in our preservation protocol as sperm showed poorer quality in non-cooled samples (0 h) than in samples stored at 15 °C for 3 and 6 h. The 24 h preservation time at 5 °C was included only as a positive control for sperm quality damage in ovine species based on previous findings of several studies [64,65,66]. In line with the results of Yániz and colleagues in sheep [67], we recorded significantly lower percentages of fast progressive and membrane-intact sperm, as well as reduced kinetic parameters and mitochondrial activity and increased apoptosis at this time compared to the 0 h. In addition, the sperm quality assessments in *Experiment 1* showed that sperm motility in terms of TM and PM improved after 6 h, with non-significant differences from the values recorded after 3 h of liquid storage. Regarding sperm functionality, viability also reached its highest value after 6 h, while apoptosis and mitochondrial activity were already optimal in the sperm preserved at 15 °C for 3 h. That is, while only two of the sperm motility and kinetic parameters studied (TM and PM) manifested an improvement, on a functionality level, the enhancement was observed in all parameters tested. This may have been due to the staining protocol used for flow cytometry evaluation, which involved the high dilution of sperm in a simple isotonic saline-based medium such as PBS. Harrison and colleagues pointed out that the dilution effect, rather than reflecting sperm viability, might correspond to an observational artefact. This is because live sperm tend to adhere to recipient surfaces in a protein-free environment, leading to a scenario where sampling from a highly diluted suspension inadvertently favors the selection of dead cells [68]. Although this tendency could affect data at all times, Ashworth et al. [69] found that ejaculated ram sperm could also perish after being washed from seminal plasma and subjected to significant dilution in a basic saline solution by destabilizing their plasma membranes due to a decrease in protective factor concentration. Supporting this theory, studies have shown that the addition of seminal plasma to highly diluted sperm notably enhances sperm viability in several species, including rabbits [70], pigs [46,71], cattle [72], and sheep [73]. Thus, high dilution rates in sperm functionality evaluation based on flow cytometry probably act as an osmotic stress test, evidencing sublethal damage to ram sperm. Moreover, these sublethal disorders seem to be reversible, as they were not detected after 3 to 6 h of semen preservation. *Experiment 2* was designed in order to identify the factor altering ram sperm quality during the first hours of liquid storage. Therefore, we replicated *Experiment 1* in ram epididymal sperm, i.e., in sperm that did not have any previous contact with seminal plasma and extender, and we established four experimental groups to test the influence of each factor separately: (1) epididymal sperm (ES), (2) epididymal sperm with seminal plasma (ES + SP), (3) epididymal sperm with INRA 96^®^ (ES + E), and (4) epididymal sperm with seminal plasma and INRA 96^®^ (ES + SP + E) simulating the ejaculate from *Experiment 1*. Contrary to the observations of Paul and colleagues [74], the supplementation of ram epididymal sperm with seminal plasma did not improve the motility and kinetic parameters. Instead, the ES + SP group showed similar results to the ES group, namely, a decrease in sperm quality over time comparable to that suffered in *Experiment 1* by the ejaculated sperm. Furthermore, the sperm of the two experimental groups containing the extender were found to have lower motility, kinetic parameters, and functionality at 0 h, which improved after 6 h at 15 °C. The addition of the extender, individually or in combination with seminal plasma, resulted in epididymal sperm behaving similarly to ejaculated sperm. According to the findings of this second experiment and considering that the adaptation time of the present study started when the samples were diluted (0 h), we assumed that extender addition could initially act as an altering factor for ram sperm regardless of origin. As observed by Herold and co-workers [75], the duration of adaptation time influences sperm stabilization intensity and the ability to maintain homeostasis and tolerance to osmotic and thermal stress. Within seminal plasma, a group of proteins known as Binder of Sperm (BSP) plays a crucial role in promoting sperm capacitation, an essential process for fertilization [76,77]. However, in the context of sperm storage, BSP proteins are harmful as they remove cholesterol and phospholipids from the sperm membrane [78]. Plante et al. [79] demonstrated in boar, stallion, and ram that casein micelles in milk and the low-density lipoprotein fraction (LDF) of egg yolk bind to free BSP, reducing the efflux of cholesterol and phospholipid from the sperm membrane during preservation. This binding process is characterized as fast, specific, saturable, and stable even after semen freezing and thawing [80], and may have occurred over the adaptation time to the extender in *Experiment 1* and in the ES + SP + E experimental group of *Experiment 2*. However, in this work, the ES + E group containing only epididymal sperm and INRA 96^®^ also needed an adaptation time to the extender before presenting optimum sperm quality, confirming that the ram sperm stabilization process is highly diluent-dependent. Thus, similarly to Anzar et al. [81] and Paul et al. [31] suggestions for the post-thawing quality of cryopreserved bull and ram sperm in an egg yolk-based extender, an extended adaptation time could also have stabilized ram sperm membranes by facilitating their coating with NPPC in all experimental groups with the extender. The latter, in turn, could have reversed the sublethal damage observed in ram sperm when quality analysis was performed close to semen collection, especially by using flow cytometry.

## 5. Conclusions

To summarize, ram sperm appear to require an adaptation time of 3 to 6 h to the extender, considered to be the altering factor, before being subjected to an accurate sperm quality assessment. This work paves the way for further investigations into ram sperm physiology during preservation and manipulation processes. In the same way, our study is interesting from a practical point of view, enabling better workflow organization in reproduction centers and an optimal expression of ram sperm attributes when cervical artificial insemination is routinely performed, linking in vitro tests and field fertility data.

## Data Availability

The data presented in this study are available on request from the corresponding author.
